# Phosphorus solubilizing microorganisms: potential promoters of agricultural and environmental engineering

**DOI:** 10.3389/fbioe.2023.1181078

**Published:** 2023-05-12

**Authors:** Chengdong Wang, Guojun Pan, Xin Lu, Weicong Qi

**Affiliations:** ^1^ Key Laboratory of Tobacco Biology and Processing, Ministry of Agriculture, Tobacco Research Institute of Chinese Academy of Agricultural Sciences, Qingdao, China; ^2^ Institute of Agricultural Resources and Environment, Jiangsu Academy of Agricultural Sciences, Nanjing, China; ^3^ School of the Environment and Safety Engineering, Jiangsu University, Zhenjiang, China; ^4^ Excellence and Innovation Center, Jiangsu Academy of Agricultural Sciences, Nanjing, China; ^5^ Key Laboratory of Saline-Alkali Soil Improvement and Utilization (Coastal Saline-Alkali Lands), Ministry of Agriculture and Rural Affairs, Nanjing, China

**Keywords:** phosphorus solubilizing microorganisms, biofertilizer, environment remediation, commercialization, cost-efectiveness, frontier research

## Abstract

Phosphate solubilizing microorganisms (PSMs) are known as bacteria or fungi that make insoluble phosphorus in soil available to plants. To date, as beneficial microbes, studies on PSMs indicated they have potential applications in agriculture, environmental engineering, bioremediation, and biotechnology. Currently high cost and competition from local microbe are the most important factors hindering PSMs commercialization and application as for instance biofertilizer, soil conditioner or remediation agent, *etc.* There are several technical strategies can be engaged to approach the solutions of these issues, for instance mass production, advance soil preparation, genetic engineering, *etc.* On the other hand, further studies are needed to improve the efficiency and effectiveness of PSMs in solubilizing phosphates, promoting plant growth, soil remediation preferably. Hopefully, PSMs are going to be developed into ecofriendly tools for sustainable agriculture, environment protection and management in the future.

## 1 Introduction

**FIGURE 1:**
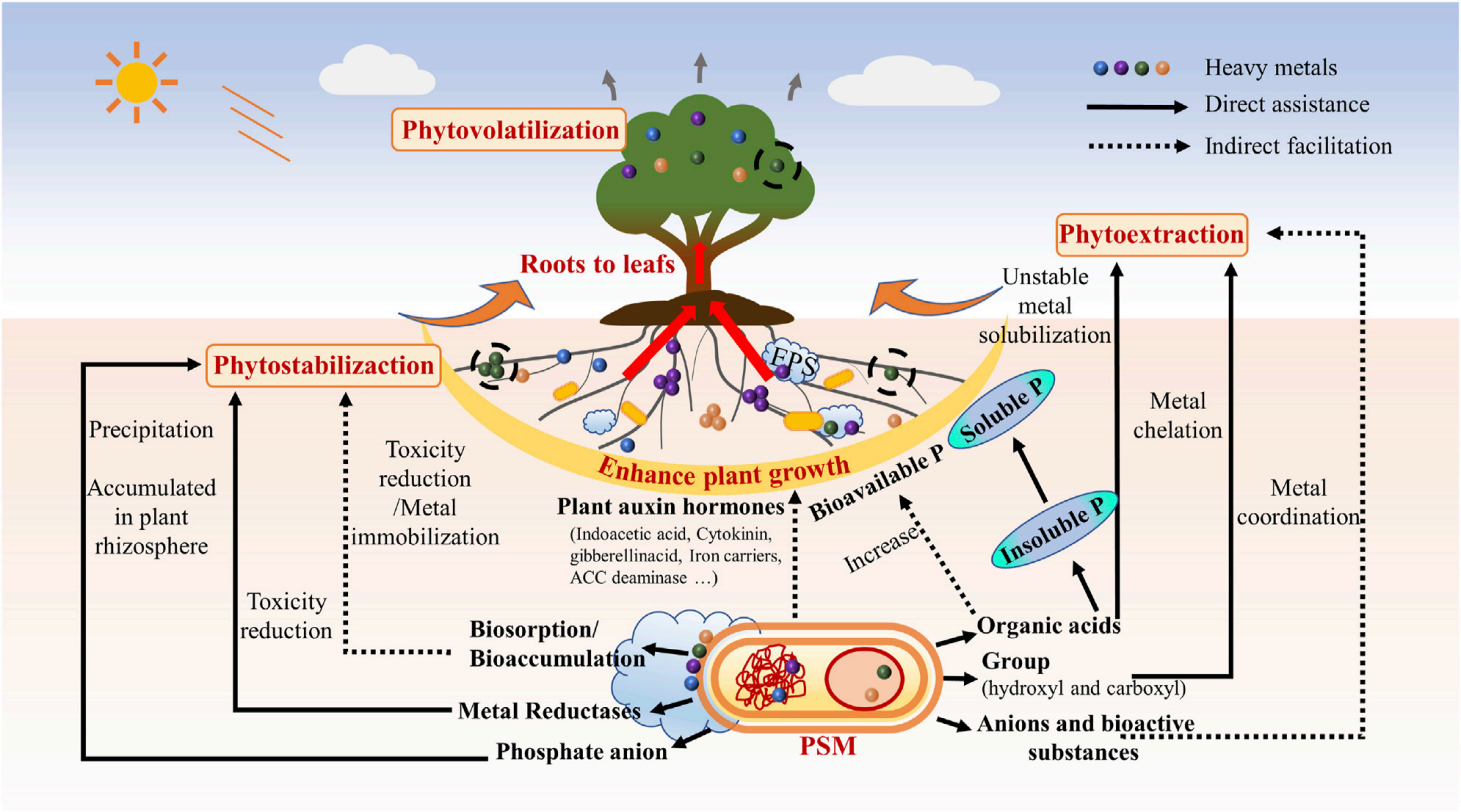
The mechanisms of phosphorus solubilizing microorganisms assisting plants nutrition absorption and soil remediation.

Phosphate solubilizing microorganisms (PSMs) are bacteria or fungi that are capable of breaking down insoluble forms of phosphorus, such as phosphates, in the soil and making it available to plants as a soluble form that can be easily absorbed (Figure 1) (Rawat et al., 2020). They are huge category of beneficial microbe including many genera for in stance *Bacillus* spp., *Pseudomonas* spp., *Streptomyces* spp., *Aspergillus* spp., *Rhizobium* spp. *Fusarium* spp., *Trichoderma* spp., *Penicillium* spp., *Serratia* spp., *Micrococcus* spp., *Stenotrophomonas* spp., *Acinetobacte*r spp., and *Agrobacterium* spp. (Rodríguez and Fraga, 1999). This process increases the overall availability of phosphorus to plants, leading to improved plant growth and crop yields. PSMs are commonly found in soil and rhizosphere, and play an important role in nutrient cycling and soil fertility (Beheshti et al., 2022). PSMs have had several applications in biotechnology. For instance, in the pharmaceutical and food industries, PSMs are used to produce antibiotics, vitamins, and other bioactive compounds (Sekurova et al., 2019); furthermore, PSMs have the potential to play a role in the development of new and efficient bioprocesses for the sustainable production of biofuels and other bio-based products (Osanai et al., 2015). The most well-known uses of PMSs are in agricultural and environmental engineering. For their outstanding nature described above, PMS are used for improving soil fertility in agriculture, enhancing plant growth and tolerance against stress of instance salinity (Luo et al., 2022), nutrient deficiency (He et al., 2022; 2021; Chen et al., 2022a) *etc.*,; and hence increasing crop yields (Chen et al., 2022b); they can also be used for bioremediation along or combining with plants, to remove for instance heavy metals from contaminated soil and water (Pinilla et al., 2008; Chen et al., 2019a).

## 2 Potential uses of PSMs in agriculture and environmental engineering

There is great potential and expectation for the future of PSMs in agriculture and environmental engineering. As the world’s population continues to grow, the demand for food and other resources will increase, putting pressure on the world’s finite resources, including phosphates. PSMs have the potential to help address this challenge by improving soil fertility and reducing the need for chemical fertilizers (Sarmah and Sarma, 2022; Tian et al., 2022).

PSMs are an emerging biotechnology tools in agriculture and environmental engineering, but the application is still in its early stages of development and not yet for commercialization. While there have been several studies demonstrating the potential of PSMs for enhancing plant growth, improving soil fertility, and detoxifying contaminated environments, much of the research is still in the laboratory or experimental stage (Chen et al., 2020; 2019b; Lai et al., 2022a).

### 2.1 For agriculture

PSMs can regulate plant metabolism by providing an increased availability of soluble phosphorus. This increased availability of phosphorus can lead to changes in plant growth and metabolism (Rodríguez and Fraga, 1999), such as an increased rate of photosynthesis, improved root development, and an enhanced ability to defend against stressors such as drought or disease (Billah et al., 2019; Kour et al., 2020). PSMs can enhance plant endogenous gene expression and help plants cope with abiotic stress by providing an increased availability of soluble phosphorus. This increased availability of phosphorus can activate signaling pathways and trigger the expression of stress-responsive genes in plants, which can enhance their ability to tolerate and resist environmental stressors such as drought, salinity, heavy metal toxicity, and temperature extremes. For example, research has shown that PSMs can increase the expression of genes involved in the regulation of water uptake, antioxidant defense, and heat shock response, which can help plants better cope with abiotic stress (Paul et al., 2017). In addition, PSMs can also stimulate the production of phytohormones such as auxins, cytokinin, gibberellins et al., which can enhance plant growth and stress tolerance (Figure 1).

### 2.2 For environmental engineering

PSMs can play a role in remediation of heavy metal pollution in soil by indirectly reducing the bioavailability of heavy metals in the soil, as showed in Figure 1. When PSMs solubilize phosphates, they create a competition for metal ions, which can reduce the uptake of heavy metals by plants and lower their concentration in the soil alone or combining with other species or materials (Lai et al., 2022b; Feng et al., 2022; Chen et al., 2023). In addition, some PSMs have been shown to produce organic acids, such as citric acid, that can chelate heavy metals and make them less available for uptake by plants (Direct assistance, Figure 1).

Another way PSMs can help remediate heavy metal pollution in soil is by promoting the growth of plants that can tolerate high levels of heavy metals. This is known as phytoremediation, and it involves using plants to remove heavy metals from the soil by absorbing and accumulating them in their tissues (Indirect facilitation, Figure 1). PSMs can help improve the growth and health of these phytoremediation plants by providing them with an available source of phosphates, which are essential for plant growth and development.

As all the function and potential use of PMS, it worth to note that here is still a need for further research to fully understand the mechanisms by which PSMs interact with plants and the environment; and to develop cost-effective and scalable methods for their application in agriculture and environmental engineering. Additionally, there are challenges associated with the large-scale production, formulation, and storage of PSMs, which must be overcome in order to make their application more practical and widespread.

The potential uses of PSMs in agriculture and environmental engineering were summarized in Table 1.

**TABLE 1:** Potential uses and benefits of applying PSMs in agriculture and environment engineering.

Agriculture	Environmental engineering
Improve soil fertility	Remove and recover phosphorus from wastewater
Enhance plant growth	Improve water quality
Increase nutrient uptake	Reduce eutrophication
Boost plant immunity	Enhance bioremediation of contaminated soils
Reduce the need for chemical fertilizers	Improve soil quality in mine reclamation
Improve crop yields	Help plants establish on degraded land
Help plants tolerate stress	Enhance microbial diversity in ecosystems
Increase soil organic matter	Mitigate the impact of agricultural runoff
Improve soil structure	Improve soil carbon sequestration
Reduce soil erosion	Increase sustainability of land use practices

## 3 The restraining factor in PSMs application and commercialization in agricultural and environmental engineering, and the promising solutions

There are several key challenges associated with the application of PSMs in crop fertilization and environmental engineering: 1) there is currently a lack of standardization in the production and application of PSMs as biofertilizers or soil remediation promoter, which can affect their efficacy and consistency; 2) PSMs are sensitive to soil conditions such as pH, salinity, and heavy metal toxicity, and may not be able to survive or function effectively in all soils; PSMs are often introduced into soil as suspensions or inoculants, but they may not persist in the soil for long periods of time, limiting their effectiveness over the long term; 3) PSMs may face competition from other microorganisms in the soil for nutrients and space, which can limit their ability to establish and grow in soil; 4) the production and application of PSMs as biofertilizers can be expensive, and the cost of this technology may be a barrier for widespread implementation in agriculture. These challenges highlight the need for further research to optimize the production and application of PSMs as biofertilizers, and to develop new and more cost-effective methods for enhancing soil fertility and crop productivity. Currently, high cost and competition from local microbe are the most important factors hindering PSMs commercialization and application.

There are several ways to reduce the cost of Phosphate Solubilizing Microorganisms (PSMs). Some of these were listed and interpreted below.

1) Mass production of PSMs: Mass production of PSMs using low-cost and efficient methods, such as fermentation, can help to reduce the cost of producing these microorganisms on a large scale.
2) Optimizing application methods: Developing more efficient and cost-effective methods for applying PSMs to soil, such as using granules (Jokkaew et al., 2022), liquid formulations (Goljanian-Tabrizi et al., 2016), or seed treatments, can help to reduce the cost of PSMs application.
3) Selecting low-cost PSMs (Lobo et al., 2019): Selecting PSMs that are naturally occurring and widely distributed, or that can be easily isolated and mass-produced, can help to reduce the cost of PSMs application.
4) Using natural sources of phosphates: Encouraging the use of natural sources of phosphates, such as rock minerals and guano, can help to reduce the need for costly commercial fertilizers.
5) Promoting the use of integrated nutrient management: Encouraging the use of integrated nutrient management practices, such as the use of cover crops, composting, and intercropping, can help to reduce the dependence on commercial fertilizers and increase the efficiency of PSMs application.

To avoid competition with local soil microorganisms for instance plant pathogens, decomposer, predatory microorganisms *et al*, the following factors should be optimized in the application of PSMs.

1) Soil preparation: To enhance the survival and persistence of PSMs, the soil should be prepared prior to their introduction. This may involve amending the soil with organic matter, adjusting the pH, or reducing soil salinity levels.
2) Inoculum preparation: The PSM inoculum should be prepared in a way that optimizes their survival and growth. This may involve selecting PSMs with high tolerance to environmental stress, such as heavy metal toxicity or salinity, or formulating the inoculum to enhance their survival and persistence in the soil (Johri et al., 1999).
3) Timing of application: PSMs should be applied at a time that minimizes competition with other soil microorganisms. For example, application during periods of low microbial activity, such as early in the growing season, may reduce competition and enhance the survival and growth of PSMs.
4) Selection of PSMs: PSMs should be selected for their ability to compete with local soil microorganisms. This may involve selecting PSMs with high competitiveness and/or selecting PSMs that are able to produce antibiotics or other metabolites that inhibit the growth of other soil microorganisms.
5) Microbial community management: The soil microbial community should be managed in a way that enhances the competitiveness of PSMs. This may involve applying compost, mulch, or other organic matter to the soil, or using other microorganisms or biostimulants to enhance the growth and competitiveness of PSMs. These factors should be optimized to ensure that PSMs are able to establish and persist in the soil, and to minimize competition with local soil microorganisms.

## 4 The current Frontier research areas in the field of PSMs

Microbial ecology: Researchers are working to understand the interactions between PSMs and other soil microorganisms, and the role that PSMs play in soil health and fertility. This research includes the study of microbial competition and cooperation, as well as the development of methods for enhancing the survival and persistence of PSMs in the soil (Nacoon et al., 2020).

Metagenomics: Metagenomic were used to approaches the diversity and function of PSMs in soil, and to identify new PSMs with enhanced abilities for solubilizing phosphates and improving soil fertility.

Biostimulants: Researches were carried out to Investigate the use of PSMs and other microorganisms as biostimulants to enhance plant growth and productivity, as well as the ability of plants to cope with environmental stress (Yahya et al., 2022).

Synthetic biology: Researchers are developing new approaches for engineering PSMs to enhance their abilities for solubilizing phosphates and improving soil fertility. This includes the use of synthetic biology to produce new strains of PSMs with improved abilities, as well as the use of CRISPR-Cas9 technology to engineer existing PSMs (Liu et al., 2020).

Soil remediation: Researchers are investigating the use of PSMs for soil remediation, with a focus on removing heavy metals and other contaminants from contaminated soils (Song et al., 2022). This research includes the development of new methods for applying PSMs to contaminated soils, as well as the study of the mechanisms by which PSMs remove contaminants from soil.

## 5 Prospect

In the future, it is expected that research into PSMs will continue to advance, with a focus on improving the efficiency and effectiveness of PSMs in solubilizing phosphates and promoting plant growth. This may involve the development of new strains of PSMs through synthetic biology and other techniques, as well as the optimization of existing PSMs for different environmental conditions and applications. In addition, it is expected that the use of PSMs will become increasingly widespread in agriculture and environmental engineering, as farmers and environmental engineers seek more sustainable and environmentally friendly solutions to the challenges posed by declining soil fertility and limited resources.

## Data Availability

The original contributions presented in the study are included in the article/Supplementary Material, further inquiries can be directed to the corresponding authors.
